# Preschool and School Meal Policies: An Overview of What We Know about Regulation, Implementation, and Impact on Diet in the UK, Sweden, and Australia

**DOI:** 10.3390/nu9070736

**Published:** 2017-07-11

**Authors:** Patricia Jane Lucas, Emma Patterson, Gary Sacks, Natassja Billich, Charlotte Elizabeth Louise Evans

**Affiliations:** 1 School for Policy Studies, University of Bristol, Bristol BS8 1TZ, UK; 2 Department of Public Health Sciences, Karolinska Institutet, SE-171 77 Stockholm, Sweden; Emma.Patterson@ki.se; 3 Centre for Epidemiology and Community Medicine, Stockholm County Council, SE-113 65 Stockholm, Sweden; 4 Global Obesity Centre, School of Health and Social Development, Deakin University, Geelong VIC 3220, Australia; gary.sacks@deakin.edu.au (G.S.); natassja.billich@deakin.edu.au (N.B.); 5 Nutritional Epidemiology Group, School of Food Science and Nutrition, University of Leeds, Leeds LS2 9JT, UK; C.E.L.Evans@leeds.ac.uk

**Keywords:** school, preshool, children, school meals, nutrition intake, policy

## Abstract

School meals make significant contributions to healthy dietary behaviour, at a time when eating habits and food preferences are being formed. We provide an overview of the approaches to the provision, regulation, and improvement of preschool and primary school meals in the UK, Sweden, and Australia, three countries which vary in their degree of centralisation and regulation of school meals. Sweden has a centralised approach; all children receive free meals, and a pedagogical approach to meals is encouraged. Legislation demands that meals are nutritious. The UK system is varied and decentralised. Meals in most primary schools are regulated by food-based standards, but preschool-specific meal standards only exist in Scotland. The UK uses food groups (starchy foods, fruit and vegetables, proteins and dairy) in a healthy plate approach. Australian States and Territories all employ guidelines for school canteen food, predominantly using a “traffic light” approach outlining recommended and discouraged foods; however, most children bring food from home and are not covered by this guidance. The preschool standards state that food provided should be nutritious. We find that action is often lacking in the preschool years, and suggest that consistent policies, strong incentives for compliance, systematic monitoring, and an acknowledgement of the broader school eating environment (including home provided food) would be beneficial.

## 1. Introduction

### 1.1. Background and Aims

There is increasing interest in policies aimed at establishing schools and Early Education and Care (EEC) as health promoting environments [1], including health education within the school curriculum, and schools as a site for healthy eating [2]. Food eaten in education and care settings makes a significant contribution to children’s total diet. Some have even suggested that a failure to provide healthy foods in schools is a breach of children’s human rights [3]. Policy, guidance, and regulation in this field has considerable potential to impact on the dietary intake of young children [4].

This paper aims to compare the school meal policies in preschool and primary educational settings in three high-income country contexts: UK, Australia, and Sweden. These were selected to illustrate the variation in OECD countries in approaches to regulation and standard setting at the regional and national level, from entirely centralised (Sweden) to entirely federal (Australia), and from highly standardised (Sweden) to highly varied (UK). We provide an overview [5] of policy and research in the field. Policy documents and regulatory tools do not lend themselves to systematic approaches, and require expert knowledge to locate the associated (grey) literature. We used our local policy knowledge to locate much of the literature included here. In addition, we searched Medline for studies published within the last 10 years in each of our target countries, with search terms for school food/meals and evaluations (the search terms are available from the corresponding author on request). The UK government is unusual in providing a searchable database of policy and government publications, and we reviewed the latest relevant policy documents using the search terms “school meals” and “school food”.

We asked: what is the current policy in each country, how many children does it reach, what do we know about how it is implemented, and what evidence exists for the impact of this approach on children’s health? We focus here on policy interventions at the national or regional level, not on research or pilot programmes.

### 1.2. School Food as a Public Health Nutrition Intervention

The diet of preschool and primary children in all three countries leaves room for improvement, with higher than recommended intakes of sugar and saturated fats [6,7], and lower intakes of fruits and vegetables [8]. Moreover, there are stark inequalities with respect to dietary quality, with lower income families reporting poorer diets [6,9,10,11,12].

The provision of meals during school hours has a long history as a public health measure [2,13]. Patterns of school food consumption mirror total diet, suggesting both that dietary choices are consistent and that school is an important eating occasion in terms of total intake [14,15]. The contribution of weekday meals for school children is approximately 30% of daily intake in both the UK [16] and Sweden [17]. There is very limited data to comment on the total contribution to diet from meals consumed in preschool [13,18].

Good quality school meals have the potential to improve children’s diets and health. The availability of healthy nutritious choices influences diet positively in school children [19]. The provision of fruit and vegetables to children in schools increases their fruit and vegetable consumption [20], and has been shown to level family differences in fruit and vegetable intakes among 11-year-old children [21]. Changes to school cafeteria environments can improve food choices at school [22]. In the earliest years, the behaviour of childcare workers themselves has a positive impact on preschool children’s intake of fruit and vegetables [23].

Health promoting schools successfully provide health education and a healthier environment for school aged children across the world [1,24,25]. Health education is an important part of the curriculum in both the primary and preschool years, and in both the UK and Sweden EEC settings are tasked to help children understand their own health choices [26,27].

### 1.3. Country Contexts

The UK has a total population of 64 million, of whom 18% are aged less than 15; 19% of Australia’s population of 24 million are children, and 17% of the 10 million people in Sweden are children [28].

Sweden is a Nordic European nation with a well-developed welfare system and relatively high taxes. Legislation is made centrally, but the 290 local authorities are responsible for the delivery of many services, including education. Preschool care/education for 1–5 year-olds is heavily subsidized (free for many or capped at ~€150 per child per month, even when privately run), and over 90% of children attend, often from 18 months of age. Primary school education covers the ages six to fifteen, is free (even when privately run), and parents incur no charges for education-related expenses, including meals.

The UK is a Western European nation. It has three devolved regions: Scotland, Wales, and Northern Ireland. National policy always applies to England, but applies to different degrees in each devolved region. Children aged 3–4 years in all regions are entitled to 15 h/week in EEC (due to rise to 30 h/week in England in September 2017); parents decide where to spend this provision from within public, private, or voluntary sector settings [29]. In England, Northern Ireland, and Scotland 2 years old in the greatest need (including those living in poverty and those with disabilities) receive the same allocation [29,30,31]. The government pays a flat rate of £55/week, which is not intended to cover food or other additional costs. Primary education is compulsory for 5–11 years old, but most children begin school aged 4 years. Primary schools may be state funded and maintained, state funded but independently run (academies, free, and church schools), or privately funded and run.

Australia is a Federation of States and Territories (New South Wales, Victoria, South Australia, Western Australia, Queensland, Tasmania, Australian Capital Territory, and Northern Territory). Education in Australia is primarily the responsibility of the states and territories. Early childhood education in Australia is not compulsory and is delivered to children through a range of settings, including childcare centres, kindergartens, and preschools in the year before full-time schooling (age 5 or 6). The Australian Government pays part of the cost of some childcare through the social security system. Compulsory education in Australia starts at around the age of five or six years. Government schools educate approximately 60% of Australian primary school students, with approximately 40% in either private or independent (including Catholic) schools.

Since policy follows organisational structures, not chronological age, we adopt the educational stage cut offs of preschool and primary school in this paper. Thus, for example, 5-year-olds attend school in England but preschool in Sweden. It is important to remember these age differences when comparing between countries. Countries apply adequacy requirements (Table 1) and limits (Table 2), which vary in their regulation, reach, cost to families, and systems of monitoring (Table 3).

## 2. UK National Preschool and Primary Food Policy

In the UK, the provision of a nutritious meal at lunch time in schools dates back over 150 years [32]. Three national school food policies apply UK-wide, while the remainder have some regional variation. Firstly, under the Nursery Milk Scheme, all children under 5 years attending EEC in all UK regions are entitled to 189 mL/day (1/3 pint) of fresh cows’ milk for free. Secondly, the Free School Fruit and Vegetable Scheme provides fruit and vegetables (3 times per week) to all state funded schools for 4–6 years old. Thirdly, Free School Meals (FSM) are provided to all children living in low income households, and since 2014, to all 4–7 year-olds in England and all 4–8 year-olds in Scotland.

### 2.1. UK Regional Preschool Food Policies

In Northern Ireland and Wales, state registered EEC settings must follow the nutritional standards set out for under 12 s (see schools below). England and Scotland have preschool-specific guidance, but while these link to compulsory standards in Scotland, in England they are voluntary.

In England, national voluntary guidelines based on four food groups (starchy foods, fruit and vegetables, non-dairy sources of protein, and dairy) apply [33] (see Table 1, Table 2 and Table 3, and Table S1). The guidance focuses on balancing intake across the day, suggesting, for instance, that lunch should contribute 30% of energy needs. Nutrient based standards are also provided [34]. For children aged 1–4 years attending full daycare, the food served should provide approximately 116 kcals, 45 g fat, and 155 g carbohydrates; no more than 34.2 Non Milk Extrinsic Sugar (NMES), 810 mg Sodium, and 2.1 g Salt; at least 14 g protein, 7.2 mg Iron, 5.7 mg Zinc, 330 mg Calcium, 390 µg Vit A, and 27 mg Vit C. The guidance makes some provision for foods brought from home (packed meals), including for meal composition, food safety guidance, and the consideration of common allergens.

The Scottish Standards follow the same nutritional guidance as the English model, and include the same food groups (see Table 1 and Table 2, and Table S1) [35]. The standards require that the provided food is “well-balanced and healthy” (p. 7) [35], and set out required portions, where a portion is “what a young child can hold in their hand” (p. 50). The standards do not cover home provided food, although additional guidance suggests food items for healthy packed lunches [35].

### 2.2. UK Regional School Food Policies

The School Food Plan introduced updated food-based standards in England in January 2014. These standards stipulate that a school meal should include an adequate provision of fruit and vegetables, dairy food, low fat proteins, and low fat starchy food (see Table 1, and Table S1). Fried foods, foods high in fats and sugars, and sweetened beverages are restricted (see Table 2). The School Food Plan applies to all state-run schools, and to those state funded but independently run Academies and Free schools created since 2015. Independent schools, along with Academies and Free schools created prior to 2015, are not covered by this policy.

There are no national policies for packed lunches, although some local government and individual schools do recommend foods to include or restrict in packed lunches, such as drinks, fruit and vegetables, and sweet and savoury snacks [36].

### 2.3. UK Reach and Implementation of Preschool and Primary Food Policy

Policy reach, cost, and monitoring is summarized in Table 3. The majority of English children now attend an EEC setting (93% 3–4 years old in 2012) [37], but we know little about how many are covered by this policy. Given part-time attendance, we do not know how many meals are eaten in EEC. Furthermore, many of these meals may be home-provided. The use of packed meals is under-recognised in preschool children, but estimates in studies have found that between 39% and 49% of Early Years Settings use only packed lunches [38,39]. We do not know whether the guidelines are adhered to in either case.

The school population is very nearly the entire child population [40], so nearly all children eat meals at school. The most recent data on the take up of school meals in England predates the introduction of universal FSM for the youngest children. At that time, 42.6% of children in primary schools ate provided meals, although it was much higher for those eligible for FSM on the basis of household income (75.1%). The average lunch cost was £2.04, and price was predictive of take up, reducing by 1.9% for every 10p more charged for the school meal [41]. The extension of FSM has high take up in Scotland (76% of children) [42].

Both primary and preschools are monitored by the Office for Standards in Education (OFSTED). Inspections should ensure standards are met where they are statutory, but not where voluntary.

### 2.4. UK Impact of Policy on Diet Quality

There is limited evidence on the quality of food provision in UK EEC, and almost none on consumption [38]. Most nurseries in England are providing meals that contain too much salt and insufficient energy [43]. There is no independent evaluation of the use of the guidelines in England nor the standards in Scotland. A before and after study conducted in England suggests that the guidelines improve staff knowledge and confidence of healthy eating guidelines, and EECs self-report a greater diversity of food and a reduced use of high sugar and salt foods [44]. However, we cannot say whether children’s diets have improved as a result of the policy in either region.

The evidence of the use of packed lunches in preschools is worrying, and probably undermines gains in provided meal quality. In one study of preschool packed lunches in the UK, 42% included crisps, 24% confectionary, and most provided a sugar-sweetened drink [38]. In the USA, preschool packed lunches are high in salt, and low in minerals, vegetables, fruit, and dietary fibre [45,46].

A number of evaluations of the primary school meal standards in the UK have demonstrated that lunchtime and whole day intake have improved with the introduction of a high quality school meal. The research mainly includes cross-sectional studies of food intake carried out after the introduction of standards and compared with intakes before the introduction of the standards [47,48,49,50]. These suggest that there may have been improvements in food provision, including increases in fruit and vegetables and reductions in non-permitted foods [51,52,53], but mixed evidence on portion size [54]. Children in schools included in Jamie Oliver’s “feed me better campaign” achieved better in standard testing relative to neighbouring schools, and authorised absences (usually for illness or planned appointments) fell [55]. A pilot trial was carried out in two areas of England looking at free meals for infants, which reported better behaviour in pupils in the classroom and healthier food at lunchtime [56].

Packed lunches in primary schools are known to be of poor quality [57], and cross-sectional studies of children across England reported that diet quality over the whole day is higher for children having a school meal compared to a packed lunch [58,59]. To the extent that meal standards and FSM extension increase school meal uptake relative to packed lunches, they therefore probably improve diet.

## 3. Swedish Preschool and Primary Meals Provision

To our knowledge, Sweden and Finland are the only countries that currently provide free school meals to all children in all years of primary school, regardless of parental income or school form. School meals are regulated by the Education Act, which states that all children attending primary school (age 6–16 years) are entitled to free and nutritious school meals [60], and by extension preschools [61]. All children are offered a prepared warm dish, salad buffet, bread, and a drink at no cost, which should be nutritionally adequate (Table 1); soft drinks are not provided (Table 2), and desserts and fried foods are rare; there are few if any vending machines, and where tuck shops exist these are often closed during lunch. Variation exists in implementation; food can be prepared on site or in an external kitchen, from scratch or from semi-processed components, and local authority or privately run.

Although not an official policy, the concept of “the pedagogic lunch” is well-established in Sweden and Finland. Teachers eat together with the children, and ideally use this opportunity to teach about food and health [62]. Preschools serve food “family style” in communal dishes to tables of approximately 10 children.

### 3.1. Swedish Preschool and Primary School Meals Guidelines

The provision of free school meals has been required by law since 1997 (Table 3), although most have done so since the 1970s, and charging for meals has not been permitted since 1946. The requirement that meals be “nutritious” (Table 1) was added in 2011, but school meals are not inspected (Table 3). The National Food Agency issues non-binding national guidelines and advice [63,64]. The guidelines focus on the whole meal experience, including quality, timing, composition, and environment. Meals are considered as more than a source of nutrition; and should be tasty, nutritious, safe, pleasant, sustainable, and integrated within the preschool/school day.

**Figure 1:**
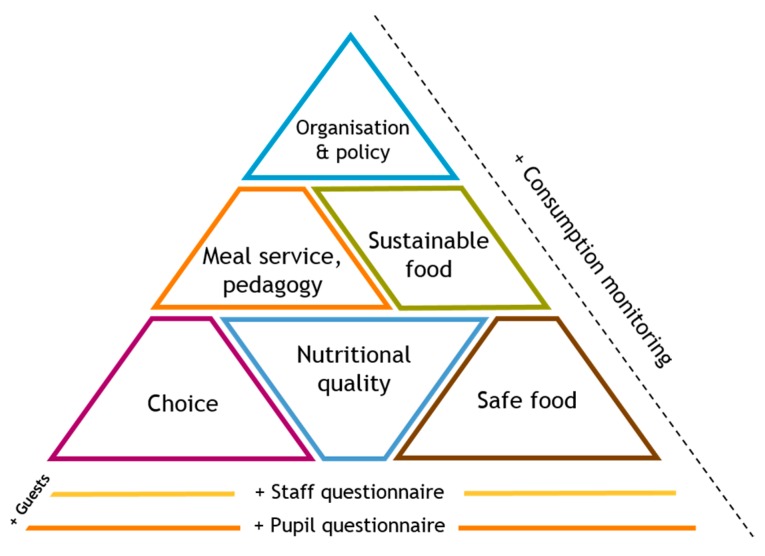
Components of SkolmatSverige instrument.

In 2010, an audit-and-feedback tool was developed by researchers and stakeholders (SkolmatSverige: School Food Sweden) to assess all of the elements of the meal experience (Figure 1) [65]. The tool aims to aid the evaluation of the impact of the 2011 law, create a nationally representative database of school meal quality, and support schools to undertake their own monitoring and evaluation and thereby improve their own school meal quality. It is web-based, free to use, and requires no training. Feedback is tailored and fully automatic.

### 3.2. Sweden: Reach, Implementation and Impact of Preschool and Primary School Meals Policy

Since policy and provision in Sweden is universal, all children in preschool/school are reached. However, there is some evidence that older children may eat lunch outside of school premises or choose to skip lunch [17]. The very fact of free provision means there are no data available to record uptake (i.e., no till or reimbursement receipts), much less to track the composition of the school meals that students choose. The nature of the traditional Swedish school lunch means that many unhealthy foods (e.g., fried foods) will simply not appear, although the implementation of the newest requirements for nutritious meals is not complete. Sociable, educational interactions modelling good eating habits have been observed in the pedagogic meal, but not among all teachers [62].

The SkolmatSverige instrument is the only current source of national data. To date, 40% of primary schools use it, but the figure is increasing [66]. In a small, but nationally representative, study of schools using the tool before and after the introduction of the law requiring “nutritious” school meals, nutritional quality increased significantly, but remained low [67]. Over a 4-week period in 2014/2015, most schools provided meals that fulfilled iron and fibre requirements (86% and 96%, respectively), but less often met requirements for vitamin D and fat (51% and 41%, respectively). Most schools (71%) offered a choice of warm meals daily, and a salad buffet with at least five components (93%) [68]. Improvements in other aspects of meal quality have not been as marked [68]. These are encouraging signs, although from a self-selecting sample.

The universal nature of Sweden’s school meal policy makes evaluation challenging because comparisons can only be historical. While the law may have had some effect, the new national guidelines, national concern about the issue, and related educational activities probably have a role too. Knowledge of the national guidelines for school meals is high [69], and three-quarters of local authorities have developed meal policies. The preliminary results from the first few years of data gathered by SkolmatSverige suggest that the repeated use of the tool results in improvements. As new data becomes available, it will be possible to validate this finding. The tool has also recently been expanded (November 2016) to include a module that helps schools measure uptake and the average amount of food that students actually consume, taking into account plate waste.

No national data on current preschool meal quality are available, and the instrument School Food Sweden was only developed for primary schools [66].

## 4. Australian Preschool and Primary School Meals

### 4.1. Australian Preschool Meal Policies

In Australia, EEC services are offered by government, community, and private providers, and are the responsibility of the states and territories (the Federal Government contributes funding to Indigenous preschool services). A National Quality Framework was agreed on by the Council of Australian Governments (COAG), and includes a National Law and Regulations that apply in all States and Territories [70]. National Quality Standards are a key element of the regulations, and apply to most forms of day care and EEC. The standards are overseen by the Australian Children’s Education and Care Quality Authority (ACEQUA), and each State and Territory is a regulatory authority with monitoring, compliance, and quality assessment roles.

Food and drink provided in EEC must comply with the legislation, regulations, and standards within the National Quality Framework. Specifically, standard 2.2 states that “healthy eating is embedded in the program for children”, and “food and drinks provided by the service are nutritious and appropriate for each child”. The meaning of “nutritious and appropriate” is not further stipulated. Each State/Territory provides guidance (see Table 1) and training to support these services to adopt nutrition and healthy eating policies. For example, the Victorian government provides a Healthy Eating Advisory Service [71], the NSW government runs the Munch & Move program [72], and the ACT has a Nutrition Support Service [73]. None of the State/Territory guidelines are mandated (Table 3).

### 4.2. Australian Primary and Secondary School Meal Policies

In Australia, most school-aged children bring their lunch from home [74,75], but the canteen or “tuckshop” plays an integral role in educating and modelling a healthy food environment [76]. The canteen in Australian schools serves as a small shop where students can purchase lunch, snacks, and drinks, and operate anywhere from one to five days per week [77]. They are operated either by canteen managers, volunteer parents, or are outsourced to external food manufacturing and supply companies.

National voluntary guidelines (based on the Australian Dietary Guidelines [78]) have been published to guide States and Territories in developing healthy school food provision policies. From these, each State/Territory has developed a set of independent healthy canteen guidelines [79,80,81,82,83,84,85,86]. Seven States and Territories have implemented mandatory standards based on their guidelines (Table 1 and Table 2).

**Figure 2:**
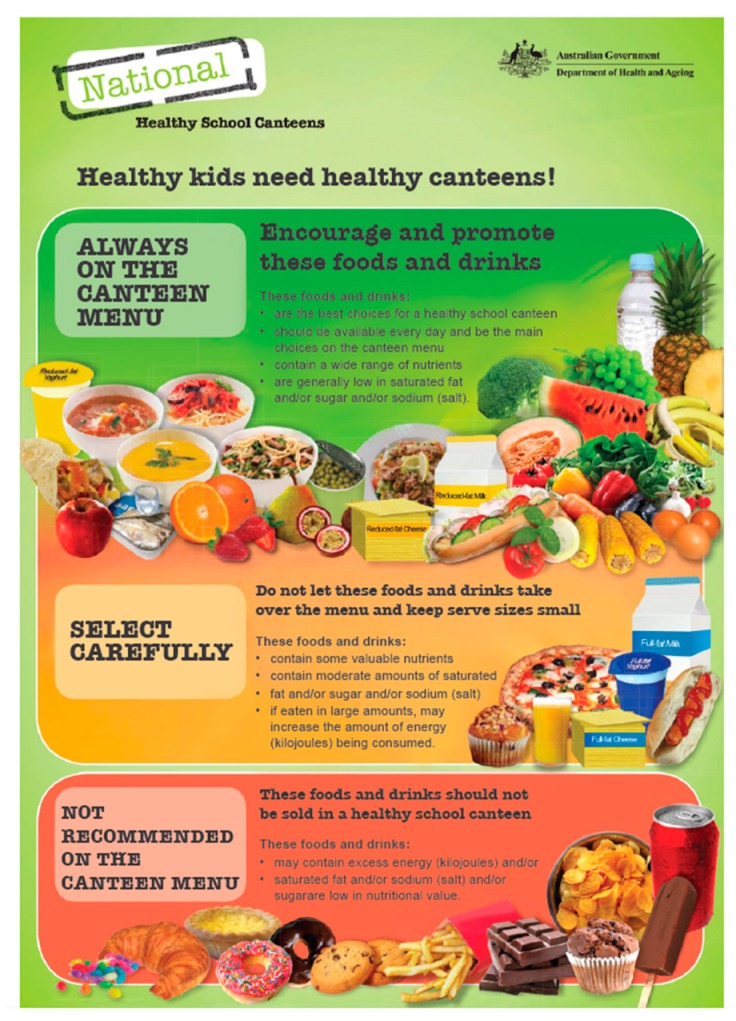
National healthy school canteens traffic light labelling guidelines. © Commonwealth of Australia 2017.

In the majority of States/Territories, the traffic light system is used in the canteen guidelines to categorise foods into “Green”, healthy foods which are encouraged (see Table 1), “Amber” (less healthy), and “Red” (least healthy) items which are discouraged (see Table 2) based on their nutritional quality. The traffic light system is relatively consistent across all States/Territories, and follows the principles outlined in the National Healthy School Canteens Traffic Light criteria (Figure 2). This system enables schools to assess their canteen menu and any other school food provision, and align food and drinks provision to these guidelines. New South Wales (NSW) has recently updated their policy in a move away from the traffic light system, to classify foods as “everyday” or “occasional”. The NSW policy mandates that even “occasional” foods are required to maintain a certain degree of healthiness (based on the government-endorsed Health Star Rating system for food labelling).

All of the other States and Territories identify “red category” foods, which are either completely banned in schools or heavily restricted (see Table 2). Guidelines are generally mandatory for government schools in each State/Territory, and are highly encouraged for Independent/Catholic schools (see Table 3).

### 4.3. Australian Reach, Implementation, and Impact of Preschool and School Meals Policies

The monitoring and compliance of policy guidelines in schools varies by State/Territory. In general, the policies are not actively enforced or routinely monitored by government. There is variable implementation across the different jurisdictions, and poor rates of adherence.

A cross-sectional study conducted by Woods et al. collected data in 2012 to assess whether schools were adhering to healthy canteen guidelines. The study explored the compliance of a convenience sample of government schools to healthy canteen guidelines, the proportion of ”Green”, “Amber”, and “Red” on each menu, and the presence of discretionary items [87]. Woods et al. found low to moderate levels of adherence to state canteen guidelines, with the highest rate of compliance in Western Australia (62% of primary and secondary schools) [87]. Four studies report low to moderate rates of compliance with government healthy canteen policies [88,89,90,91]. The self-reported implementation of guidelines was demonstrated to be high in one Queensland study [92]. However, Principal and canteen manager self-reporting has been demonstrated to be in poor agreement with the gold standard of compliance assessment [93]. A recently published randomised controlled trial further found that a menu audit and feedback system made only moderate impacts on compliance, although some improvements were seen [94].

Since most school children bring lunch from home, evidence about the content of these and any spillover effect for the canteen guidelines is needed.

Similarly, there is very limited evidence about food provided or eaten in preschool settings. Since the standard is simply that food is ”nutritious”, monitoring compliance is not particularly meaningful. One qualitative study suggests that the guidelines are not used [95]. Furthermore, due to the highly disparate nature and uptake of EEC services in Australia, it is difficult to know how many children are using preschool provision and how many meals are eaten there.

## 5. Discussion

Quite different approaches to ensuring school meal quality are taken in the three countries reviewed here. Sweden has almost universal provision and uptake of school meals from preschool through primary years, and a requirement for nutritious meals, but this requirement is not strictly monitored and the guidelines issued for the meals are non-binding. In contrast, Australia largely relies on home-provided meals in the primary years supplemented by in-school canteens. National “traffic light” guidance for canteen food in primary schools exists, but implementation is at a state level and adherence is poor. Preschools in Australia have state-specific support for the provision of healthy food, but national standards simply stipulate food and drinks provided must be “nutritious and appropriate”, which makes this standard too vague to be enforceable. The UK uses food-based standards in both the primary and preschool years, but these are only statutory in some contexts. Free school meal uptake is high, but where meals are paid for (including in preschools), the use of home provided meals is common, undermining the positive contribution of food standards to diet. In none of the three countries are the mechanisms for the monitoring of these food standards clearly enforced, limiting our knowledge and potentially limiting impact on diet and health. In both the UK and Australia, there is little regulation and provision for the youngest children.

In the UK, researchers have been active in using opportunistic research designs to estimate the impact of policy changes in the primary years. The introduction of food-based standards for primary school meals combined with an increased uptake following the introduction of universal free meals in the infant years appears to have had positive impacts on children’s diet.

The range and breadth of additional guidance and supporting materials available are a strength of the current policy approaches [26,33,71,72,73]. The wealth of information supplied particularly to preschools is valued by some providers and parents [44], and has the potential to influence diet outside of school too. The ambition to integrate food into the pedagogic environment in Sweden, and to a lesser extent in the UK’s preschools, is also a strength. Evidence suggests that embedding discussions about healthy eating and exposing children to healthy food choices is likely to be useful in shaping their long term eating preferences [13,96].

Two core weakness emerge, however. The first is that provided school meals are only part of the picture of food consumed during school hours. Packed lunches commonly provide alternate meals in both Australia and the UK, but additional food sources in all countries include vending machines, tuck shops, bake sales, other foods brought from home, and food provided in care before and after school. As children age, they may also be leaving school premises to purchase food from local food retailers. The nutritional advantages of high quality meal provision are undermined where few children take up this offer and/or where food from other sources is not included in healthy eating plans.

Secondly, all three countries appear to have weak mechanisms for monitoring compliance with healthy eating policies, and lack provisions for monitoring children’s actual intake. This means that they are unable to comment with certainty on the extent to which schools provide food as envisaged in the policy, and are unable to assess whether policy changes have resulted in improved nutritional intake. Importantly, we also cannot comment on whether these initiatives counteract health inequalities. The voluntary self-monitoring system in Sweden could enable a comparison of school meal quality between catchment areas of differing socio-economic position in the future, but its use is not widespread enough for this to be the case yet. Where free meals are provided for the most disadvantaged (as is the case among older primary aged children in the UK), nutritionally sound meals have the potential to decrease inequalities, but policy complexity makes this difficult to assess. In England, a particular oddity of the policy evolution is that the youngest children are not included in free meal provision, so although the most disadvantaged children are preferentially provided with free preschool places, their meals are neither free nor covered by school food standards. Anecdotally, we note that some nurseries are providing free meals to the most deprived 2 years old from within their own budget, but this micro-level solution is neither testable nor scalable.

### 5.1. Implications for Policy

The improvement of school food through national or state nutritional guidelines or standards is ongoing in many countries [2,13,97]. We believe that these policies are likely to be important for the long-term health of our populations through their influence on dietary intake and food habits. Two systematic reviews of methods to increase fruit and vegetable intake in children [20,98] suggest the best evidence for success is for provision through schools.

The implications for school meal policies are that: (a) enforcement of policy is necessary to see improvements; (b) uptake of provided food is crucial to deliver benefits; and (c) monitoring of uptake, nutritional intake, and differential intake by social groups is needed to demonstrate whether provided school food is, in fact, a public health success. Others have reached similar conclusions [4], particularly considering the value of monitoring and evaluation for policy success [99]. Overly burdensome data collection can interfere with policy implementation [51], but light touch regulation, using existing education inspection mechanisms, that requires schools to report on their compliance with guidelines and their uptake of provided food could achieve much with little additional effort. In addition, mechanisms to provide clear accountability for compliance with standards, and strong incentives for compliance (e.g., tied to budget mechanisms, or individual Principal performance assessments) need to be explored. The Swedish self-monitoring tool is working well to encourage quality improvements, and it provides a model for efficient audit.

We also need whole-school approaches to healthy eating which are broader than school meals. This should include the consideration of meals and snacks brought from home, but also wider actions to address attitudes towards healthy foods. Banning adults (staff and visitors) from smoking or using tobacco products on the premises or at any school-related activities is a key feature of tobacco free schools [100]. Adopting similar “whole school” approaches to junk-food free schools may be appropriate.

Population-level improvements in health need population-level responses, and political and policy actions addressing the food environment are needed [101]. Reductions in the extensive marketing of unhealthy food in children’s immediate environment (fast food outlets and convenience stores) [102], and increases in the relative price of unhealthy compared to healthy food are needed [103], and would support schools in their efforts.

### 5.2. Implications for Practice

Canteen-style provision now dominates in primary schools in all three countries, but the pedagogic model used in Sweden deserves greater attention [62]. The use of school meal times as an opportunity to talk about food and food choices may be valuable [104], but only if used by teachers [62]. Although further research is needed, we would consider this to be areas of promising practice.

Similarly, involving parents and carers in changes in school food may promote the generalisability of change [20,105]. Food policies that focus only on safety aspects and the avoidance of allergens are unlikely to engage parents as partners in improving diet. Top down policy has little effect if it is not coupled with, and sensitive to, local implementation [106].

### 5.3. Implications for Research

We know of few published high quality longitudinal studies or randomised controlled trials evaluating the impact of school meal policies on nutrition behaviour, diet quality, and health [107,108,109,110,111], although some are currently underway [112,113,114]. A more rigorous evaluation of the benefits of school meals on diet quality and health outcomes (such as obesity), including longitudinal studies or randomised controlled trials, are needed to assess the impact of policies, particularly on reducing inequalities in diet and health.

Much of the practice outlined in this paper has not been monitored or evaluated. While guidelines themselves are often based on extensive and expert review of nutritional needs in children, the evidence to support the implementation of such guidelines is regrettably lacking. The research needs are many in this field, but we propose the most urgent ones here: (a) the exploitation of routinely collected data to comment on the impact of policy roll-out when innovation occurs; (b) research on the implementation of nutritional and food-based guidelines; (c) research into the contribution of foods consumed during school hours to total diet across the preschool and primary years; and (d) research into the long term effects of healthy school meal provision on diet and educational achievement. Core to this would be (e) the introduction of standard measures and indicators to assess food intake [97].

## 6. Conclusions

There is good evidence to suggest that meals eaten in the preschool and primary years should be a target for improving children’s dietary habits and preferences. In the three countries reviewed, action in the preschool years generally lags behind schools, and policies tend to lack enforceability. Policies are needed which have clear standards, systems for monitoring compliance and reach, and which acknowledge the whole school eating environment including home provided meals. While important, school food policies will have limited impact in the absence of broader public health and political action to improve our food environment.

## Figures and Tables

**Table 1:** School food adequacy standards in the UK, Australia, and Sweden.

Food	Standard or Guidance	Level, Country (Jurisdiction)
Fruit and vegetables	Minimum number portions per day/meal	School and Preschool, UK (England, Scotland)
Meat, fish, eggs, and non-dairy protein		
Starchy foods	Recommended number of portions per day	School and Preschool, UK (England, Scotland)
Milk and dairy		
Drinks	Fresh water to be freely available	School and Preschool, UK (England, Scotland)
	Diluted fruit juice or fresh cows’ milks	Preschool, UK (England, Scotland)
Traffic Light: “Green” foods	Minimum of 60% of food and snacks sold should be “Green”	School, Australia (WA *)
	Should be available every day the canteen is open	School, Australia (NT, SA, Q, V *)
Every-day (Core) foods	Recommended to make up 75% of food and drinks sold	School, Australia (NSW *)
“Nutritious” meals	Meals must be “nutritious”. Preschool meals must also be “varied”, and “evenly distributed over the day”	School and Preschool, Sweden
“Good meals in preschool”	Suggestions provided for meal components and nutritional content (e.g., serve fatty fish twice within 4 weeks, serve only milk and water to drink, provide a wide selection of salads, include wholegrain versions of products etc.)	School and Preschool, Sweden
“Good school meals”		

* Northern Territory (NT); South Australia (SA); Queensland (Q); Victoria (V); New South Wales (NSW); Western Australia (WA).

**Table 2:** School food limiting standards in the UK, Australia, and Sweden.

Food	Standard or Guidance	Level, Country (Jurisdiction)
Meat, fish, eggs, and non-dairy protein	Maximum number portions	School, UK (England), Preschool UK (Scotland)
Starchy foods	Maximum number of occasions higher fat versions offered (fried starchy foods and cheese as protein)	School, UK (England)
Milk and dairy food		
Traffic Light: “Amber” foods	Maximum of 40% of foods and drinks sold should be “Amber”	School, Australia (WA *)
	“Amber” foods should be limited and sold in smaller serve sizes	School, Australia (NT, SA, Q, V *)
Desserts & Puddings	To be served only once per day with main meal	School and Preschool, UK (England, Scotland)
Occasional (non-core) foods	Maximum of 25% of food and drinks sold should be “occasional” and least healthy not sold	School, Australia (NSW *)
Foods high in fat, salt, and sugar	Avoided or limited	School and Preschool, UK (England, Scotland)
Traffic Light: “Red” foods	“Red” foods prohibited or limited to special occasions (no more than twice per term)	School, Australia (NT, SA, Q, V *)
	“Red” foods prohibited	School, Australia (NT, SA, Q, V *)
Salt and condiments	Reduced or not salt in cooking, No salt available to add. Condiments avoided or size limited.	School and Preschool, UK (England, Scotland)
	Should not be available to students	School and Preschool, Sweden
Drinks	All those not specified to be excluded	School and Preschool, UK (England, Scotland)
	“Amber” drinks should be avoided and sold in smaller serve sizes	School, Australia (NT, SA, Q, V *)
	“Red” drinks (sugary) not to be sold and not permitted	School, Australia (all states and territories)
	No sweet drinks containing sugar or sweetener	School and Preschool, Sweden
Confectionary	Not permitted	School and Preschool, UK (England, Scotland)
	Not permitted	School, Australia (all states and territories)
	Should not be provided	School and Preschool, Sweden

* Northern Territory (NT); South Australia (SA); Queensland (Q); Victoria (V); New South Wales (NSW); Western Australia (WA).

**Table 3:** School food policy regulation and monitoring in the UK, Sweden, and Australia. Abbreviation: EEC, early education and care.

Policy	Country (Jurisdiction)	Level	Reach	Cost to Families	System of Monitoring
Food Based Standards for Schools in England	UK (England)	School	Mandatory for local government controlled schools and schools that became academies before 2010 and after June 2014. Recommended for remaining academies and private schools.	Free for 4–7 years	Included in the Office of Standards in Education (OFSTED) inspection report on healthy eating.
				Paid for other years	
Voluntary Guidance	UK (England)	Preschool	Voluntary	Paid	Not monitored in OFSTED inspections, although the Early Years Foundation Stage (EYFS) requires that provided food must be “healthy balanced and nutritious” (EYFS 2017).
Preschool standards	UK (Scotland)	Preschool	Mandatory for all settings registered with the Care Inspectorate.	Paid	Inspected by the Care Inspectorate. EEC must show they provide opportunities for children to learn about diet and health. EEC must show children have access to a well-balanced and healthy diet. For example, through use of a food policy.
Traffic light food groups	Australia (Australia Capital Territory)	School	Policy applies to all food services activities within a school setting. Mandatory for government schools. In independent and Catholic schools, it is not mandatory but highly recommended.	Paid	Independent compliance monitoring. Free assessment, resources and training available for canteens. Facilitate government license agreements with school canteens.
	Australia (Northern Territory)	School	Policy applies to all food services activities within a school setting. Mandatory for government schools	Paid	Independent compliance monitoring, schools responsible for oversight.
	Australia (South Australia)	School	Policy applies to all food services activities within a school setting. Mandatory for government schools. Catholic and independent school sectors support implementation of the policy.	Paid	Independent compliance monitoring, schools responsible for oversight. Resources provided to canteens.
	Australia (Queensland)	School	Policy applies to all food services activities within a school setting. Mandatory for government schools.	Paid	Independent compliance monitoring, schools responsible for oversight. Training and resources provided to canteens.
	Australia (Tasmania)	School	Schools are recommended (not mandatory) to apply to have their canteen accredited.	Paid	Training, resources and monitoring available to canteens.
	Australia (Victoria)	School	Policy applies to all food services activities within a school setting. Mandatory for government schools.	Paid	Independent compliance monitoring, schools responsible for oversight. Optional monitoring and assessment of canteens available. Training and resources provided to canteens.
Every-day vs. occasional foods	Australia (New South Wales)	School	Policy applies to all food services activities within a school setting. Mandatory for government schools. Catholic and independent schools are also encouraged to participate.	Paid	Independent compliance monitoring, schools responsible for oversight. Resources provided to canteens.
Traffic light and core vs. non-core foods	Australia (Western Australia)	School	Policy applies to all food services activities within a school setting. Mandatory for government schools. Catholic schools have a similar mandatory policy.	Paid	Principals are required to develop and implement a whole school-based policy on the provision of healthy food and drinks and ensure that the school canteen/food service menu complies with the requirements of the policy.
Nutritious preschool meals	Sweden	Preschool	Legislation. Mandatory for all (whether local authority or privately run).	Free or subsidized *	Monitoring of provision and cost is not required. Monitoring of nutritional quality falls to the management (i.e., the local authority or owner).
Nutritious school meals	Sweden	School	Legislation. Mandatory for all (whether local authority or privately run).	Free	Monitoring of provision and cost is not required. Monitoring of nutritional quality falls under the School Inspectorate, but is delagated to the school management (i.e., the local authority or owner).
“Good meals in preschool”, “Good school meals”	Sweden	Preschool, School	Guidelines. Voluntary for all.	Free or subsidized *	Not applicable, but for schools, compliance with several of the guidelines can be demonstrated using “School Food Sweden” tool.

* Some parents pay a capped low fee for preschool; meals are provided for all.

## Supplementary Materials

The following are available online at www.mdpi.com/2072-6643/9/7/736/s1. Table S1: School Food Standards in England, and Preschool Standards for England and Scotland.
