# Supplementary material for: Preschool and School Meal Policies: An Overview of What We Know about Regulation, Implementation, and Impact on Diet in the UK, Sweden, and Australia
Source: Nutrients. 2017 Jul 11;9(7):736. doi: 10.3390/nu9070736 (PMC5537850; doi:10.3390/nu9070736)
Supplement: Supplementary file 1 [file nutrients-09-00736-s001.zip › nutrients-195173-supplementary.pdf]

Table S1: School Food Standards in England, and Preschool Standards for England and Scotland

**Food Group**	Food Based Standards for School in England	Guidance for Preschools in England	Standards for Preschools in Scotland
**Fruit and vegetables**	At least one portion of fruit and one portion of vegetables or salad (40g) provided per day (e.g., fruit-based desserts, or casseroles and stews containing vegetables).	Five portions each day; a portion with each main meal and with some snacks. Examples include fresch, frozen, canned, dried and juiced fruit and vegetables and pulses.	A main meal shall provide a minimum of two servings of food from the fruit and vegetable group, excluding fresh fruit juice. At least one serving must be vegetables.<br>A light meal shall provide at least one serving of fruit or vegetables.
**Meat, fish, eggs and other non-dairy sources of protein**	A portion of protein rich food should be provided each day including red meat at least twice per week, and fish at least once a week. Oily fish should be provided at least once every three weeks. Non-dairy, iron-rich sources of protein (eggs, beans, pulses, soya products) provided for non-meat eaters and at least twice a week for all children.<br>Processed meat or chicken products may only be served once per week.	Two portions each day; provided in lunch and tea (evening meal). Vegetarian children should have two or three portions. Examples include meat, poultry, fish, shellfish, eggs, meat alternatives, pulses, nuts	A food from this group shall be provided as part of every main or light meal (excluding breakfast).<br>Avoid or limit meat products and highly processed foods (e.g. sausages, pies, bought beef burgers) to a maximum of once a week.<br>Oily fish shall be provided once every week.
**Starchy food**	A portion of low fat starchy food should be provided daily, e.g. bread, potatoes, pasta, rice and other grains. Fried versions (chips, fried rice) should not be available on more than two days in any week. Bread with no fat or oil added should also be provided daily.	4 portions/day; one in each meal and as part at least one snack. Examples include bread, potatoes, starchy root vegetables, pasta, rice.	For both main and light meals, at least one serving from this group shall be provided as part of every meal.

**Milk and dairy food**	A portion of dairy food should be available daily, e.g. cheese or yoghurts. Reduced fat milk for drinking should also be available. A dish containing cheese should not be served as the only protein option more than twice each week.	Three portions each day; provided as part of any meal, snack or drink. Examples include milk, cheese, yoghurt, fromage fraise, custard puddings made from milk	Whole milk should be offered to children under the age of 2. Additional guidance that young children need 3 servings of dairy per day
**Desserts & Puddings**	n/a	The guidance suggests that puddings should be included with main meals as they can provide energy and "essential nutrients such as calcium and iron" (p.24).	Included in meal descriptions. Confectionary is not allowed.
**Foods high in fat, salt and sugar**	No confectionery[1], chocolate and chocolate-coated products or savoury snacks (e.g. crisps) should be provided throughout the school day. Nuts, seeds, fruit[2] or vegetables with no added salt, sugar or fat are permitted. Provision of cakes, puddings and biscuits should be limited to lunch times. Savoury crackers or breadsticks can only be served with fruit or vegetables or dairy food as part of a school meal.	Guidance states that fried foods, pastries and processed meat should be limited. Salty foods should be avoided, and sugary foods should be limited.	Oils that are high in polyunsaturated and/or monounsaturated fats shall be used. Spreadable fats (with a fat content of more than 70%) that are high in polyunsaturated and/or monounsaturated fats shall be used. Spreads with a fat content of less than 70% fat should not be used. Deep frying should not be used as a cooking method. Products that have been deep fried during the manufacturing process, such as fishcakes, should be limited to once per week and on different days to ensure that children attending on

			the same day each week are offered variety.<br>No savoury snacks shall be provided as part of the meal or snack.<br>The daily maximum sodium intake for 1-3 year olds is given as 0.8g and 1.2 g for 4-6 year olds
**Drinks**	There should be easy access at all times to free, fresh drinking water.<br>The other drinks that can be provided are:<br>● Plain water (still or carbonated).<br>● Reduced fat milk or fortified soya milk.<br>● Fruit juice or vegetable juice.<br>● Plain yoghurt drinks<br>● Unsweetened combinations of fruit or vegetable juice with:<br>○ Plain water<br>○ Reduced fat/soya milk or yoghurt (with or without plain water)<br>● Tea, coffee, hot chocolate<br><br>All of these drinks should not exceed 5% added sugars/honey and should be available as individual portions not exceeding 300mls (for fruit juice and fruit juice drinks, the individual portion must not exceed 150ml).	Drinks should be only milk or water between meals, diluted fruit juice may be offered with meals.	The only drinks that can be provided as part of a young child's meals are:<br>• milk – whole milk for children aged 1–2 years. Semi-skimmed milk can be provided for children age 2–5, if eating well.<br>• plain water<br>• a combination of pure unsweetened fruit juiceor plain water (50:50 dilution) and limited to mealtimes only.<br>Children should be offered a choice of milk and plain water to drink during the day.

**Salt and condiments**	No product should exceed the maximum salt content specified in the prevailing Responsibility Deal salt targets or other target nutrient specifications. No salt shall be available to add to food after the cooking process is complete. Condiments may be available only in sachets.	No salt should be added to food during cooking or eating. Salty condiments condiments should be avoided.	No salt shall be added to food while cooking.<br>No salt or condiments, for example soy sauce, shall be available to add to food after the cooking process is complete.<br>Ready-to-use cooking sauces are frequently high in added sugar and salt and are not recommended as a regular ingredient in meals provided for young children.

[1] Including chocolate, biscuits containing or coated with chocolate, cereal bars, processed fruit bars, sweets
[2] Dried fruit is permitted to have 0.5% fat as a glazing agent.
